# Clinical Impact of the Transient Return of Spontaneous Circulation Before Extracorporeal Cardiopulmonary Resuscitation in Patients with Refractory Out-of-Hospital Cardiac Arrest: A Nationwide Observational Study

**DOI:** 10.3390/jcm15103584

**Published:** 2026-05-07

**Authors:** Haksun Kim, Jae-Guk Kim, Gu-Hyun Kang, Yong-Soo Jang, Wonhee Kim, Hyun-Young Choi, Chiwon Ahn

**Affiliations:** 1 Department of Emergency Medicine, Kangnam Sacred Heart Hospital, College of Medicine, Hallym University, Seoul 07441, Republic of Korea; haksun93@gmail.com (H.K.); emkang@hallym.or.kr (G.-H.K.); amicoys@hallym.or.kr (Y.-S.J.); wonsee02@hallym.or.kr (W.K.); chy6049@hallym.or.kr (H.-Y.C.); 2 Department of Emergency Medicine, College of Medicine, Chung-Ang University, Seoul 06974, Republic of Korea; cahn@cau.ac.kr

**Keywords:** extracorporeal cardiopulmonary resuscitation, outcome, out-of-hospital cardiac arrest

## Abstract

**Background/Objectives:** Out-of-hospital cardiac arrest (OHCA) remains associated with poor survival despite advances in resuscitation. Extracorporeal cardiopulmonary resuscitation (ECPR) is a potential salvage therapy for refractory OHCA, but optimal patient selection remains uncertain. Transient return of spontaneous circulation (ROSC) before ECPR has been proposed as a prognostic marker, although its clinical significance remains unclear. This study aimed to evaluate the impact of transient ROSC before ECPR initiation on survival and neurological outcomes. **Methods:** We conducted a nationwide retrospective cohort study based on the Korean Out-of-Hospital Cardiac Arrest Surveillance registry (2016–2022). The study population was divided according to the occurrence of transient ROSC prior to ECPR. The primary endpoint was survival to hospital discharge, while favorable neurological outcome served as the secondary endpoint. Propensity score matching (PSM) and multivariable logistic regression analyses were performed. **Results:** After PSM, survival to hospital discharge (16.7% vs. 17.6%; *p* = 1.000) and favorable neurological outcomes (10.8% vs. 8.8%; *p* = 0.814) did not differ between groups. In multivariable analysis, transient ROSC was not associated with survival before (AOR 1.179; 95% CI 0.564–2.464; *p* = 0.662) or after PSM (AOR 0.592; 95% CI 0.206–1.698; *p* = 0.329). Similarly, no association was observed for favorable neurological outcomes before (AOR 1.246; 95% CI 0.526–2.951; *p* = 0.617) or after PSM (AOR 1.094; 95% CI 0.307–3.900; *p* = 0.890). **Conclusions**: Transient ROSC before ECPR initiation was not associated with improved survival or neurological outcomes in patients with refractory OHCA.

## 1. Introduction

Out-of-hospital cardiac arrest (OHCA) is a major cause of mortality worldwide, with survival rates consistently below 10% despite advancements in resuscitation science [1,2]. For patients with refractory OHCA, defined as those who fail to achieve sustained return of spontaneous circulation (ROSC) after conventional cardiopulmonary resuscitation (CPR), extracorporeal CPR (ECPR) has emerged as a promising salvage therapy [3,4,5]. ECPR provides temporary circulatory support and allows time to address the reversible causes of cardiac arrest [6,7]. However, the decision to initiate ECPR remains complex and must balance resource intensity with patient benefits.

A growing body of evidence suggests that transient ROSC—brief episodes of spontaneous circulation prior to ECPR cannulation—may serve as an important prognostic marker in refractory OHCA [8,9,10]. Transient ROSC is defined variably across studies but is often characterized by the presence of a palpable pulse or measurable perfusion lasting less than 20 min before ECMO initiation. Several recent studies have demonstrated that even a brief restoration of circulation may enhance cerebral perfusion and mitigate ischemic injury, potentially priming patients for improved neurological outcomes after ECPR initiation [9,11,12].

In a secondary analysis of patients with ECPR-treated OHCA, Otani et al. [9] reported that transient ROSC before ECMO was independently associated with favorable neurological recovery. Similarly, a recent multicenter study by Kim et al. further substantiated that patients who achieved transient ROSC before ECMO cannulation had significantly higher survival rates than those who did not receive pre-ECMO perfusion [11]. These findings align with prior registry-based evidence indicating that ROSC events, even if unsustained, may represent a patient subgroup with higher intrinsic recoverability or more rapidly reversible etiologies [12,13].

Despite these insights, the prognostic significance of transient ROSC remains underexplored in large cohorts and has not been integrated into the ECPR candidacy criteria. Understanding whether transient ROSC confers a neuroprotective advantage or simply reflects early resuscitation quality is essential for refining inclusion strategies and optimizing patient outcomes. Moreover, the interplay among pre-hospital variables, low-flow duration, and transient ROSC in the context of ECPR deployment warrants further investigation.

In this study, we aimed to evaluate the impact of transient ROSC before ECPR initiation on both clinical outcomes, including survival and neurological status, among patients with refractory OHCA undergoing ECPR.

## 2. Methods

### 2.1. Study Design and Data Source

A retrospective cohort analysis was conducted based on the Korean Out-of-Hospital Cardiac Arrest Surveillance (OHCAS) registry, which provides nationwide population-level data. The registry comprehensively collects data on all OHCA cases managed by emergency medical services (EMS), incorporating both pre-hospital and in-hospital information. Oversight of the registry is provided by the Korea Centers for Disease Control and Prevention (KCDC), which ensures data quality through systematic audits and validation processes conducted by trained medical reviewers. The registry was designed in accordance with internationally accepted Utstein-style guidelines and incorporates elements of the Resuscitation Outcomes Consortium protocols, ensuring consistency with global cardiac arrest research standards. However, several clinical variables were not captured and were therefore excluded from the analysis, including (1) hemodynamic parameters during and after ROSC; (2) biochemical markers, such as serum lactate and arterial blood gas measurements; and (3) detailed characteristics of transient ROSC, including its duration and frequency.

The study protocol was approved by the Institutional Review Board of Kangnam Sacred Heart Hospital (IRB No. HKS 2025-12-038). Data access was authorized by the Korea Disease Control and Prevention Agency in 2025. The requirement for informed consent was waived due to the retrospective nature of the study and the use of de-identified data. The study’s methodological approach aligned with the guidelines stipulated in the Strengthening the Reporting of Observational Studies in Epidemiology checklist for observational studies.

### 2.2. Study Population

This study included patients recorded in the OHCAS registry between January 2016 and December 2022. Among 217,356 patients with OHCA, we excluded those younger than 18 years of age, those whose arrest was caused by non-medical causes such as trauma, drowning, or intoxication, and those who had sustained ROSC before ECPR initiation. Additional exclusion criteria included documented do-not-resuscitate orders, death upon arrival at the hospital, lack of ECPR implementation, and missing outcome data. After applying these criteria, we selected adult patients with OHCA who underwent ECPR at the emergency department (ED) for refractory cardiac arrest. Patients younger than 18 years were excluded from restricting the analysis to an adult population. No upper age limit was applied, as age alone is not considered an absolute contraindication for ECPR in clinical practice [7,14], and decisions regarding ECPR initiation are typically individualized.

According to the registry, ECPR was performed in a selected subset of patients with refractory OHCA, based on institutional protocols and the attending physician’s clinical judgment. In general, ECPR candidacy was considered for patients with a presumed reversible etiology and favorable prognostic features, such as witnessed arrest and shorter no-flow or low-flow durations. However, the registry does not provide detailed information regarding the specific reasons for the non-performance of ECPR in individual cases.

### 2.3. Variable Definitions

The demographic and clinical variables included age, sex, witnessed arrest status, and administration of bystander CPR. We recorded the initial cardiac rhythm, presence of rhythm conversion, and pre-existing comorbidities, such as hypertension, diabetes mellitus, chronic kidney disease, respiratory diseases, and dyslipidemia, based on documented medical history (Table S1). Cardiac arrest was presumed to occur when conditions such as ischemic heart disease, arrhythmia, or cardiac tamponade were present.

In-hospital interventions included reperfusion therapy, which was defined as the administration of intravenous thrombolysis or percutaneous coronary intervention (PCI). Targeted temperature management (TTM) was performed according to institutional protocols, employing either surface or intravascular cooling systems with automated temperature control. Devices such as Arctic Sun® (Medivance Corp., Louisville, KY, USA)and CoolGard 3000® (Alsius Corp., Irvine, CA, USA) were commonly used. TTM was initiated with a target temperature of 32 to 36 °C and maintained for 12 to 24 h in accordance with American Heart Association guidelines.

Transient ROSC was defined as a temporary return of spontaneous circulation, characterized by the presence of a palpable pulse or measurable blood pressure lasting more than 1 min but resolving within 20 min prior to ECMO initiation during both pre-hospital and in-hospital resuscitation, as documented by EMS providers and hospital clinicians. However, the registry does not consistently distinguish the exact location of occurrence.

ROSC was assessed as part of routine resuscitation care by trained emergency medical service personnel in the pre-hospital setting and by emergency physicians in the emergency department. Circulatory status was evaluated intermittently during rhythm checks or when clinically indicated, based on the presence of a palpable pulse, measurable blood pressure, and available monitoring parameters. The registry captures the occurrence of ROSC but does not provide detailed information on the duration, frequency, or hemodynamic quality of transient ROSC episodes.

Low-flow time was defined as the duration from the initiation of CPR to the establishment of extracorporeal circulation. Transient ROSC prior to ECPR initiation was not considered the termination of the low-flow state [6]. For witnessed cardiac arrest cases in which the exact time of collapse was not documented, the EMS call time was used as the starting point for the time interval. When both the EMS call time and witnessed collapse time were available, the earlier of the two was used to determine the time interval.

The interval from call or witnessed arrest to ED arrival was defined as the time from the emergency call (or witnessed arrest time, whichever was earlier) to ED arrival. The ED-to-ECPR interval was defined as the time from ED arrival to the establishment of extracorporeal circulation (ECMO pump-on), rather than the initiation of cannulation.

Neurological status was assessed according to the Cerebral Performance Categories (CPC) scale, where CPC scores of 1–2 were defined as favorable and scores of 3–5 as unfavorable (Table S2).

### 2.4. Outcome Measures

The primary endpoint was survival to hospital discharge, while the secondary endpoint was favorable neurological status at discharge, defined as a CPC score of 1–2.

### 2.5. Statistical Analysis

We first performed descriptive analyses to compare baseline characteristics between patients with and without transient ROSC. Categorical variables are presented as frequencies and percentages and were compared using the chi-square or Fisher’s exact tests. Continuous variables are described using means with standard deviations or medians with interquartile ranges and compared using Student’s *t*-test or the Mann–Whitney U test, depending on the distribution. Missing data were handled using complete-case analysis, as the proportion of missing values was minimal (<5%) and deemed unlikely to introduce significant bias [15,16].

To minimize confounding, we performed propensity score matching (PSM) in both transient and non-transient ROSC groups. PSM was conducted in a 1:1 ratio using nearest-neighbor matching without replacement, applying a caliper of 0.2 standard deviations of the logit-transformed propensity score. Covariates included in the propensity score model comprised age, sex, witnessed arrest, bystander CPR, presumed cardiac etiology, initial shockable rhythm, underlying comorbidities, use of mechanical CPR, and post-cardiac arrest interventions, including reperfusion therapy and TTM. Covariate balance between the matched groups was evaluated using standardized mean differences (SMDs), with values <0.1 indicating negligible imbalance.

Although some cardiac arrests were witnessed, the exact time of arrest was not consistently recorded in the registry. Therefore, time-related variables were not included in the propensity score model due to limitations in accurately defining total low-flow duration. The registry does not directly provide the interval from CPR initiation to the establishment of extracorporeal circulation, and available time metrics may reflect heterogeneous pre-hospital and in-hospital processes. Accordingly, these variables were adjusted for multivariable regression analyses.

Following PSM, we performed multivariate logistic regression analyses in the overall cohort before PSM and the propensity score-matched cohort to evaluate whether transient ROSC before ECPR initiation independently influenced clinical outcomes before and after PSM.

Variables were selected for inclusion based on clinical relevance and prior evidence. Multivariable logistic regression models were then developed using a stepwise backward elimination approach, incorporating clinically pertinent covariates. The following covariates were entered into the initial model: age, sex, bystander CPR, witnessed arrest, presumed cardiac etiology, initial shockable rhythm, hypertension, diabetes mellitus, heart disease, chronic kidney disease, stroke, respiratory disease, mechanical CPR, reperfusion therapy, TTM, time from call or witness to ED arrival, and time from ED arrival to ECPR. Adjusted odds ratios (AORs) with corresponding 95% confidence intervals (CIs) were estimated to assess independent associations.

To evaluate potential effect modification by delays in ECPR initiation, subgroup analyses were conducted according to the median ED-to-ECPR interval. Multivariable logistic regression models were applied within each subgroup to assess the association between transient ROSC and clinical outcomes. All analyses were performed using two-sided tests, with a *p*-value of <0.05 considered statistically significant. Statistical computations were carried out using SPSS (version 26.0; IBM Corp., Armonk, NY, USA) and R (version 3.3.2; R Foundation for Statistical Computing, Vienna, Austria).

## 3. Results

### 3.1. Patient Characteristics

**Figure 1:**
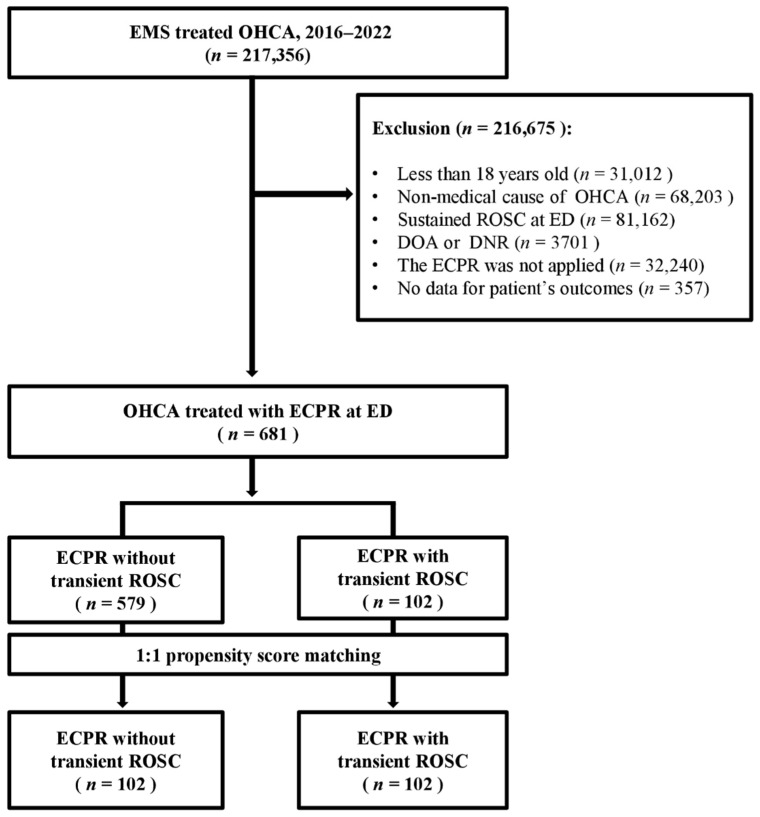
Study flow diagram illustrating patient inclusion and exclusion. EMS, emergency medical system; OHCA, out-of-hospital cardiac arrest; ROSC, return of spontaneous circulation; ED, emergency department; DOA, dead on arrival; DNR, do not resuscitate; ECPR, extracorporeal cardiopulmonary resuscitation.

From January 2016 to December 2022, a total of 217,356 adult patients with out-of-hospital cardiac arrest were transported to hospitals nationwide in South Korea. Of these, only 681 patients (0.31%) were included in the final analysis after applying the study inclusion criteria underscoring the highly selected nature of patients undergoing ECPR (Figure 1). Patients were stratified according to the presence of transient ROSC prior to ECPR initiation into two groups: those without transient ROSC (*n* = 579) and those with transient ROSC (*n* = 102). Baseline characteristics and clinical outcomes for each group are summarized in Table 1.

The proportion of male patients was similarly high in both groups (83.3% vs. 83.8%; *p* = 1.000), and median age did not differ significantly (54.0 [44.0–64.0] vs. 57.0 [46.0–65.0] years; *p* = 0.532). Regarding pre-hospital factors, bystander CPR was less frequently performed in patients with transient ROSC than in those without transient ROSC (56.9% vs. 68.2%; *p* = 0.033). The rate of witnessed arrest was similar between the two groups (83.3% vs. 82.6%; *p* = 0.961), and the presumed cardiac etiology did not differ significantly (90.2% vs. 94.3%; *p* = 0.177). The proportion of initial shockable rhythm was comparable between the two groups (22.5% vs. 23.8%; *p* = 0.877). Regarding comorbidities, hypertension tended to be less common in the transient ROSC group (27.5% vs. 38.2%; *p* = 0.050), whereas the prevalence of diabetes, heart disease, respiratory disease, chronic kidney disease, and stroke did not differ significantly between the groups. Mechanical CPR was used less frequently in patients with transient ROSC (23.5% vs. 34.4%; *p* = 0.042). The rates of post-cardiac arrest care prior to ECPR showed no statistically significant differences, although reperfusion therapy tended to be less common among those with transient ROSC (56.9% vs. 67.2%; *p* = 0.056). The use of TTM was similar between the groups (13.7% vs. 17.3%; *p* = 0.459). The time intervals from collapse or witnessed arrest to ED arrival were comparable (42.0 min in both groups; *p* = 0.621). However, patients with transient ROSC experienced a significantly longer interval from ED arrival to ECPR initiation (87.0 [53.0–110.0] vs. 79.0 [40.0–99.0] min; *p* = 0.034).

In-hospital outcomes, including survival to hospital discharge (16.7% vs. 17.4%; *p* = 0.961) and a favorable neurological outcome (CPC 1–2) at discharge (10.8% vs. 10.5%; *p* = 1.000), were not significantly different between the groups.

### 3.2. Changes in Baseline Characteristics and Time Intervals After PSM

Before PSM, mechanical CPR was more frequently used in the no transient ROSC group than in the transient ROSC group (34.4% vs. 23.5%; *p* = 0.042). After matching, no significant difference was observed between the groups (*p* = 1.000).

After PSM, the interval from ED arrival to ECPR initiation remained significantly longer in the transient ROSC group compared with the no transient ROSC group (87.0 [53.0–110.0] vs. 78.0 [38.0–96.0]; *p* = 0.032).

### 3.3. Survival and Neurological Outcome at Hospital Discharge After PSM

Changes in absolute SMD and dot plots of absolute SMD among patients undergoing ECPR are shown in Figure S1. A comparison of outcomes between the matched groups is summarized below.

Survival to hospital discharge (16.7% vs. 17.6%; *p* = 1.000) and favorable neurological outcomes with CPC 1–2 (10.8% vs. 8.8%; *p* = 0.814) did not differ between the matched groups (Table 2). Both unmatched and matched analyses consistently demonstrated that transient ROSC before ECPR initiation did not improve survival or neurological outcomes in patients with refractory OHCA who received ECPR.

### 3.4. Multivariable Analysis of Survival and Neurological Outcomes at Hospital Discharge Using Logistic Regression Before and After PSM

**Figure 2:**
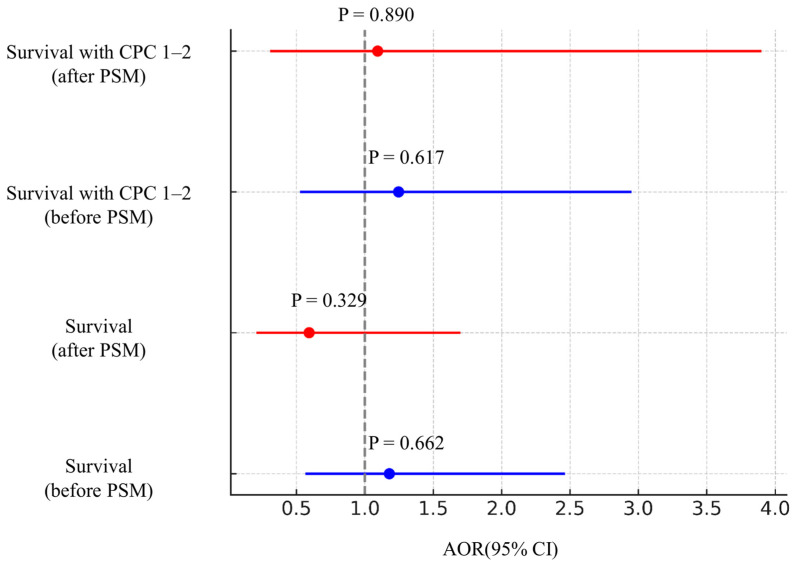
Outcomes at hospital discharge were analyzed using multivariable logistic regression before and after PSM. PSM, propensity score matching; AOR, adjusted odds ratio; CI, confidence interval.

Multivariate logistic regression analyses were performed to evaluate whether transient ROSC before ECPR initiation independently influenced clinical outcomes before and after PSM (Table 3 and Figure 2).

#### 3.4.1. Survival to Hospital Discharge

In the unmatched cohort, transient ROSC did not independently predict survival to discharge (AOR 1.179; 95% CI 0.564–2.464; *p* = 0.662). After PSM, transient ROSC remained non-significant and was not associated with better odds of survival (AOR 0.592; 95% CI 0.206–1.698; *p* = 0.329) (Table 3 and Figure 2).

#### 3.4.2. Favorable Neurological Outcome

Similarly, transient ROSC showed no significant association with favorable neurological outcomes before matching (AOR 1.246; 95% CI 0.526–2.951; *p* = 0.617). In the matched cohort, this association remained non-significant (AOR 1.094; 95% CI 0.307–3.900; *p* = 0.890) (Table 3 and Figure 2).

In both analytical models, unmatched and propensity score-matched, transient ROSC consistently failed to demonstrate any statistically significant or clinically meaningful association with improved outcomes.

### 3.5. Subgroup Analysis of Survival and Neurological Outcomes According to Median ED-to-ECPR Intervals Using Multivariable Logistic Regression Before and After PSM

Subgroup analyses were performed to evaluate whether the association between transient ROSC and survival to hospital discharge differed according to the ED-to-ECPR interval before and after PSM (Table 4).

#### 3.5.1. Survival to Hospital Discharge Stratified by Median ED-to-ECPR Interval

Before PSM, transient ROSC was not significantly associated with survival in either the shorter (<80.5 min; AOR 1.744, 95% CI 0.640–4.752; p = 0.277) or longer (≥80.5 min; AOR 1.329, 95% CI 0.318–5.553; p = 0.696) ED-to-ECPR interval groups.

After PSM, no significant association was observed between transient ROSC and survival in the shorter ED-to-ECPR interval group (<80 min; AOR 0.891, 95% CI 0.187–4.254; *p* = 0.885). In the longer interval group (≥80 min), the adjusted odds ratio suggested a lower likelihood of survival in the transient ROSC group; however, this did not reach statistical significance (AOR 0.118, 95% CI 0.012–1.127; *p* = 0.063) (Table 4).

#### 3.5.2. Favorable Neurological Outcome Stratified by Median ED-to-ECPR Interval

For favorable neurological outcomes, transient ROSC was not significantly associated with improved outcomes in either subgroup.

Before PSM, no significant association was observed in both the shorter (<80.5 min; AOR 1.495, 95% CI 0.476–4.694; *p* = 0.491) and longer (≥80.5 min; AOR 1.094, 95% CI 0.318–3.900; *p* = 0.890) ED-to-ECPR interval groups.

Similarly, after PSM, transient ROSC remained unassociated with favorable neurological outcomes in both the shorter (<80 min; AOR 1.801, 95% CI 0.254–12.761; *p* = 0.556) and longer (≥80 min; AOR 0.497, 95% CI 0.027–17.744; *p* = 0.827) ED-to-ECPR interval groups (Table 4).

**Figure 3:**
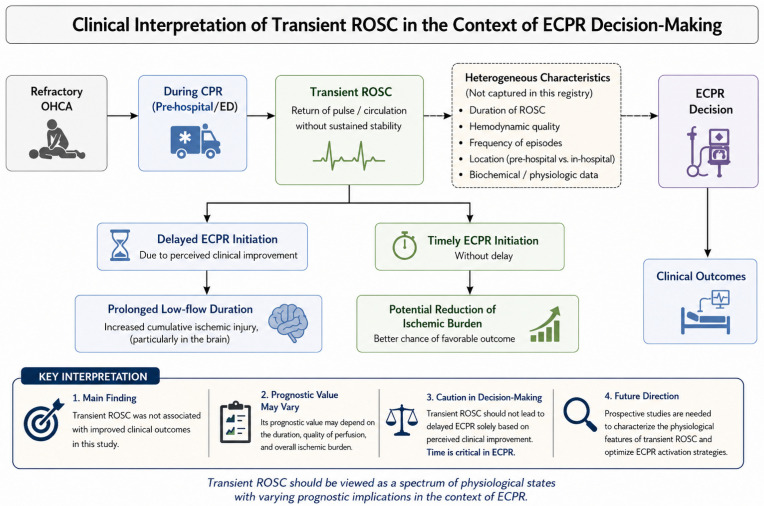
Conceptual framework for the clinical interpretation of transient ROSC in ECPR-treated OHCA. ROSC, return of spontaneous circulation; ECPR, extracorporeal cardiopulmonary resuscitation; OHCA, out-of-hospital cardiac arrest; CPR, cardiopulmonary resuscitation.

In both the primary multivariable logistic regression analyses and subgroup analyses stratified by median ED-to-ECPR intervals, transient ROSC was not independently associated with improved survival to hospital discharge or favorable neurological outcomes among patients undergoing ECPR (Figure 3).

## 4. Discussion

In this multicenter cohort, transient ROSC before ECPR initiation, as defined in this study, was not associated with improved survival or neurological outcomes. However, this finding should be interpreted with caution, as transient ROSC may not necessarily reflect meaningful physiological recovery.

Transient ROSC likely represents brief and hemodynamically unstable periods of circulation that are insufficient to substantially reduce cumulative ischemic injury or obviate the need for extracorporeal support. Accordingly, clinical decisions regarding ECPR candidacy should not be delayed or modified based solely on transient ROSC. Moreover, post-cardiac arrest physiology is characterized by cerebral microcirculatory dysfunction and systemic hemodynamic instability such that transient restoration of measurable circulation may not translate into sustained end-organ perfusion [6,17,18]. Therefore, transient ROSC has limited value, which can be interpreted as the resolution of the low-flow state in ECPR candidates.

Although Otani et al. [9] reported an association between transient ROSC before ECPR and improved neurological outcomes, we did not observe a similar benefit. This discrepancy may be partly explained by differences in the overall ischemic burden and variations in ROSC definitions. In the study by Otani et al. [9], the duration of CPR before ECPR initiation was substantially shorter (approximately 50–60 min), whereas our cohort exhibited markedly prolonged resuscitation times exceeding 100 min, which may have attenuated the potential physiological impact of transient circulatory recovery. Furthermore, in the study by Otani et al. [9], transient ROSC was defined as a detectable pulse or measurable blood pressure persisting for longer than 1 min, which may encompass a heterogeneous mixture of both sustained and non-sustained ROSC episodes. In contrast, in our study, transient ROSC was defined using a shorter duration threshold (<20 min), representing a conceptually distinct clinical condition. However, we were unable to quantify the duration or hemodynamic quality of transient ROSC episodes, precluding differentiation between brief, unstable pulses and more sustained periods of effective perfusion. As the prognostic significance of transient ROSC is likely context-dependent—determined by its duration, perfusion quality, and cumulative ischemic burden, the lack of such granular data may have introduced heterogeneity and misclassification. Consequently, the aggregation of physiologically diverse transient ROSC episodes may have obscured clinically meaningful differences and attenuated any potential association with outcomes. These findings suggest that the absence of a significant effect may reflect limitations of a binary definition of transient ROSC rather than a true lack of biological relevance, highlighting the need to consider transient ROSC as a spectrum of physiological states in the context of ECPR.

Previous ECPR studies have consistently demonstrated that outcomes are strongly time-dependent, emphasizing the importance of minimizing low-flow duration and optimizing the timing of extracorporeal support. Shoji et al. [19] reported that minimizing low-flow duration was correlated with improved survival and neurological outcomes, underscoring the time-sensitive nature of ECPR implementation. Similarly, Supady et al. highlighted that the potential benefits of ECPR depend on its implementation within an optimized system of care to limit prolonged low-flow exposure [14,19]. In this context, transient ROSC may provide a misleading signal of restored circulation despite persistent myocardial dysfunction or imminent rearrest. Although not statistically significant, a notable finding of this study is the longer ED-to-ECPR interval observed in patients with transient ROSC, suggesting that transient ROSC may lead to delayed ECPR initiation due to perceived clinical improvement. Given that ECPR outcomes are highly time-dependent, such delays may prolong low-flow duration and increase cumulative ischemic injury, particularly in the brain. Consequently, this extended ischemic burden may attenuate any potential benefit of transient perfusion and bias the observed association toward a null effect.

Transient ROSC does not guarantee adequate cerebral perfusion. Even when measurable blood pressure is temporarily achieved, the cerebral perfusion pressure may remain insufficient, particularly in the presence of severe metabolic and hemodynamic instability [20,21]. Consequently, despite appearing clinically reassuring, transient ROSC may not meaningfully modify the pathophysiological mechanisms that drive neurological injury.

These suggest that the prognostic significance of transient ROSC may be context-dependent and influenced by both the cumulative duration of ischemia and the operational criteria used to define ROSC events.

Despite the application of PSM and multivariable adjustment, the possibility of residual confounding remains. The registry lacks several critical variables that may have influenced both treatment allocation and clinical outcomes, such as CPR quality, granular physiological parameters, and the nuances of clinician decision-making. Furthermore, unmeasured system-level factors—including pre-hospital logistics, institutional workflows, and the immediate availability of the ECMO team—could have contributed to the observed variability in ECPR initiation times. Consequently, these uncaptured factors may have influenced the associations reported in this study, necessitating a cautious interpretation of the results.

### Limitations

This study has several limitations. First, the study population represents a highly selected subgroup of OHCA patients, with less than 0.5% of registry cases undergoing ECPR, which may limit generalizability. Second, the registry does not include laboratory variables such as serum lactate or other markers of perfusion, limiting adjustment for ischemic severity and introducing potential residual confounding. Third, we were unable to determine the location of transient ROSC (pre-hospital vs. emergency department), which may influence its clinical interpretation. Fourth, the registry lacks detailed information on the duration and hemodynamic quality of transient ROSC, potentially introducing misclassification bias and obscuring differences between brief, ineffective perfusion and more sustained circulatory recovery. Finally, the relatively prolonged ED-to-ECPR intervals and the absence of a direct measure of total low-flow duration further limit interpretation, as cumulative ischemic burden is a key determinant of outcome and may have attenuated any potential benefit of transient ROSC.

## 5. Conclusions

Transient ROSC, as defined in this study, was not associated with improved clinical outcomes. However, its prognostic value may depend on the duration, quality of perfusion, and overall ischemic burden, which were not fully captured in this analysis. Further prospective studies are warranted to clarify the physiological characteristics of transient ROSC and refine ECPR activation strategies.

## Figures and Tables

**Table 1:** Characteristics of the study population according to the presence of transient ROSC before ECPR initiation at ED.

Variables	Total	No Transient ROSC ^a^	Transient ROSC ^a^	*p*
	(*N* = 681)	(*N* = 579)	(*N* = 102)	
Sex, male, *n* (%)	570 (83.7%)	485 (83.8%)	85 (83.3%)	1.000
Age, years [median (IQR)]	56.0 [46.0–64.0]	57.0 [46.0–65.0]	54.0 [44.0–64.0]	0.532
Bystander CPR	453 (66.5%)	395 (68.2%)	58 (56.9%)	0.033
Witnessed arrest	563 (82.7%)	478 (82.6%)	85 (83.3%)	0.961
Cardiac origin	638 (93.7%)	546 (94.3%)	92 (90.2%)	0.177
Initial shockable rhythm	161 (23.6%)	138 (23.8%)	23 (22.5%)	0.877
Pre-existing comorbidity, *n* (%)				
HTN	249 (36.6%)	221 (38.2%)	28 (27.5%)	0.050
DM	165 (24.2%)	143 (24.7%)	22 (21.6%)	0.579
Heart disease	135 (19.8%)	117 (20.2%)	18 (17.6%)	0.643
Respiratory disease	19 (2.8%)	18 (3.1%)	1 (1.0%)	0.380
CKD	20 (2.9%)	20 (3.5%)	0 (0.0%)	0.112
Stroke	23 (3.4%)	20 (3.5%)	3 (2.9%)	1.000
Mechanical CPR	223 (32.7%)	199 (34.4%)	24 (23.5%)	0.042
Post-cardiac arrest care				
Reperfusion treatment ^b^	447 (65.6%)	389 (67.2%)	58 (56.9%)	0.056
TTM	114 (16.7%)	100 (17.3%)	14 (13.7%)	0.459
Time interval, min				
Call or witness to ED arrival *	42.0 [23.0–74.0]	42.0 [23.0–74.0]	42.0 [22.0–71.0]	0.621
From ED arrival to ECPR ^$^	80.5 [43.0–99.0]	79.0 [40.0–99.0]	87.0 [53.0–110.0]	0.034
Outcome at hospital discharge				
Survival to discharge	118 (17.3%)	101 (17.4%)	17 (16.7%)	0.961
Survival with CPC 1–2	72 (10.6%)	61 (10.5%)	11 (10.8%)	1.000

Abbreviations: ROSC, return of spontaneous circulation; ECPR, extracorporeal cardiopulmonary resuscitation; ED, emergency department; IQR, interquartile range; CPR, cardiopulmonary resuscitation; DM, diabetes mellitus; HTN, hypertension; CKD, chronic kidney disease; TTM, targeted temperature management. ^a^ Transient ROSC was defined as a return of spontaneous circulation lasting >1 min but <20 min prior to ECPR initiation. ^b^ Reperfusion therapy included intravenous thrombolysis and percutaneous coronary intervention. * Time interval from EMS call or witnessed arrest to arrival at the ED. ^$^ Time interval from ED arrival to initiation of ECPR.

**Table 2:** Characteristics of the study population according to the presence of transient ROSC after propensity score matching.

Variables	Total	No Transient ROSC ^a^	Transient ROSC ^a^	*p*
	(*N* = 204)	(*N* = 102)	(*N* = 102)	
Sex, male, *n* (%)	168 (82.4%)	83 (81.4%)	85 (83.3%)	0.854
Age, years [median (IQR)]	55.0 [45.0–63.0]	55.5 [45.0–62.0]	54.0 [44.0–64.0]	0.970
Bystander CPR	116 (56.9%)	58 (56.9%)	58 (56.9%)	1.000
Witnessed arrest	172 (84.3%)	87 (85.3%)	85 (83.3%)	0.847
Cardiac origin	184 (90.2%)	92 (90.2%)	92 (90.2%)	1.000
Initial shockable rhythm	46 (22.5%)	23 (22.5%)	23 (22.5%)	1.000
Pre-existing comorbidity, *n* (%)				
HTN	55 (27.0%)	27 (26.5%)	28 (27.5%)	1.000
DM	38 (18.6%)	16 (15.7%)	22 (21.6%)	0.369
Heart disease	26 (12.7%)	8 (7.8%)	18 (17.6%)	0.059
Respiratory disease	1 (0.4%)	0 (0.0%)	1 (0.9%)	1.000
CKD	0 (0.0%)	0 (0.0%)	0 (0.0%)	1.000
Stroke	6 (2.9%)	3 (2.9%)	3 (2.9%)	1.000
Mechanical CPR	49 (24.0%)	25 (24.5%)	24 (23.5%)	1.000
Post-cardiac arrest care				
Reperfusion treatment ^b^	116 (56.9%)	58 (56.9%)	58 (56.9%)	1.000
TTM	34 (16.7%)	20 (19.6%)	14 (13.7%)	0.348
Time interval, min				
Call or witness to ED arrival *	36.0 [20.0–71.0]	32.0 [20.0–70.5]	42.0 [22.0–71.0]	0.369
From ED arrival to ECPR ^$^	80.0 [47.0–101.0]	78.0 [38.0–96.0]	87.0 [53.0–110.0]	0.032
Outcome at hospital discharge				
Survival to discharge	35 (17.2%)	18 (17.6%)	17 (16.7%)	1.000
Survival with CPC 1–2	20 (9.8%)	9 (8.8%)	11 (10.8%)	0.814

Abbreviations: ROSC, return of spontaneous circulation; ECPR, extracorporeal cardiopulmonary resuscitation; ED, emergency department; IQR, interquartile range; CPR, cardiopulmonary resuscitation; DM, diabetes mellitus; HTN, hypertension; CKD, chronic kidney disease; TTM, targeted temperature management. ^a^ Transient ROSC was defined as a return of spontaneous circulation lasting >1 min but <20 min prior to ECPR initiation. ^b^ Reperfusion therapy included intravenous thrombolysis and percutaneous coronary intervention. * Time interval from EMS call or witnessed arrest to arrival at the ED. ^$^ Time interval from ED arrival to initiation of ECPR.

**Table 3:** Multivariable logistic regression analysis for outcomes at hospital discharge before and after propensity score matching.

	Before PSM		After PSM	
	AOR (95%CI)	*p*	AOR (95%CI)	*p*
Survival with CPC 1–2 *				
Transient ROSC (−)	1		1	
Transient ROSC (+)	1.246 (0.526–2.951)	0.617	1.094 (0.307–3.900)	0.890
Survival to hospital discharge *				
Transient ROSC (−)	1		1	
Transient ROSC (+)	1.179 (0.564–2.464)	0.662	0.592 (0.206–1.698)	0.329

Model of multivariate logistic regression analysis is stepwise backward elimination. PSM, propensity score matching; CPC, Cerebral Performance Categories; ROSC, return of spontaneous circulation; AOR, adjusted odds ratio; CI, confidence interval. *Adjusted odds ratios for age, sex, bystander CPR, witnessed cardiac arrest, cause of cardiac arrest, initial shockable rhythm, diabetes mellitus, hypertension, heart disease, chronic kidney disease, stroke, respiratory disease, mechanical CPR, reperfusion therapy, targeted temperature management, time interval from call or witness to ED arrival, and time interval from ED arrival to ECPR.

**Table 4:** Subgroup analysis of outcomes according to median ED-to-ECPR intervals using multivariable logistic regression before and after propensity score matching.

Before PSM				
Median time interval	<80.5 min		≥80.5 min	
	AOR (95%CI)	*p* value	AOR (95%CI)	*p* value
Survival to hospital discharge *				
Transient ROSC	1.744(0.640–4.752)	0.277	1.329(0.318–5.553)	0.696
Survival with CPC 1–2 *				
Transient ROSC	1.495(0.476–4.694)	0.491	1.094(0.307–3.900)	0.890
After PSM				
Median time interval	<80 min		≥80 min	
	AOR (95%CI)	*p* value	AOR (95%CI)	*p* value
Survival to hospital discharge *				
Transient ROSC	0.891(0.187–4.254)	0.885	0.118(0.012–1.127)	0.063
Survival with CPC 1–2 *				
Transient ROSC	1.801(0.254–12.761)	0.556	0.697(0.027–17.744)	0.827

Model of multivariate logistic regression analysis is stepwise backward elimination. PSM, propensity score matching; CPC, Cerebral Performance Categories; ROSC, return of spontaneous circulation; AOR, adjusted odds ratio; CI, confidence interval. * Adjusted odds ratios for age, sex, bystander CPR, witnessed cardiac arrest, cause of cardiac arrest, initial shockable rhythm, diabetes mellitus, hypertension, heart disease, chronic kidney disease, stroke, respiratory disease, mechanical CPR, reperfusion therapy, targeted temperature management, and time interval from call or witness to ED arrival.

## Data Availability

The authors used a database made available by the Korea Disease Control and Prevention Agency, which holds authority over the OHCA registry dataset in Korea. Access to this dataset requires permission and interested parties can request access through the official website (https://www.kdca.go.kr/injury/biz/injury/main/mainPage.do) (accessed on 10 January 2026).

## Supplementary Materials

The following supporting information can be downloaded at: https://www.mdpi.com/article/10.3390/jcm15103584/s1, Figure S1: Changes in absolute standardized mean differences (A) and dot plot of absolute standardized mean differences (B) in ECPR patients before and after propensity score matching; Table S1: Definition and detailed classification of pre-existing comorbidity; Table S2: Glasgow-Pittsburgh Cerebral Performance Categories (CPC) scores and their corresponding descriptions.

## Abbreviations

The following abbreviations are used in this manuscript:

CPC	Cerebral Performance Categories
CPR	cardiopulmonary resuscitation
ECPR	extracorporeal cardiopulmonary resuscitation
ED	emergency department
EMS	emergency medical services
IRB	Institutional Review Board
KCDC	Korea Centers for Disease Control and Prevention
OHCA	out-of-hospital cardiac arrest
OHCAS	Out-of-Hospital Cardiac Arrest Surveillance
PCI	percutaneous coronary intervention
PSM	propensity score matching
ROSC	return of spontaneous circulation
TTM	targeted temperature management
