# Supplementary material for: Clinical Impact of the Transient Return of Spontaneous Circulation Before Extracorporeal Cardiopulmonary Resuscitation in Patients with Refractory Out-of-Hospital Cardiac Arrest: A Nationwide Observational Study
Source: J Clin Med. 2026 May 7;15(10):3584. doi: 10.3390/jcm15103584 (PMC13207045; doi:10.3390/jcm15103584)
Supplement: Supplementary file 1 [file jcm-15-03584-s001.zip › Supplementary materials_Transient ROSC Before ECPR.pdf]

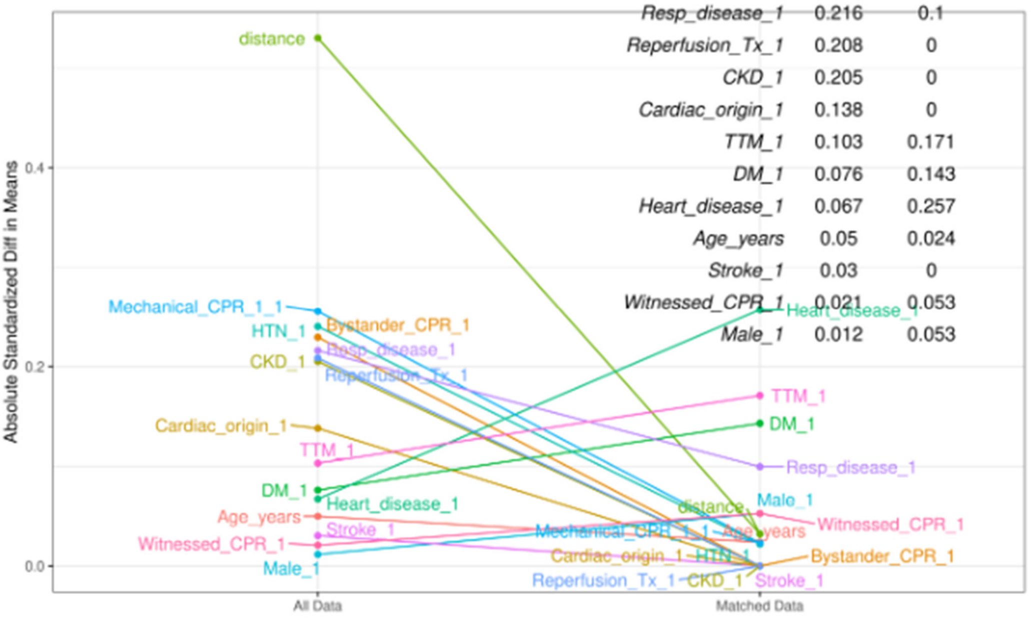

(A)

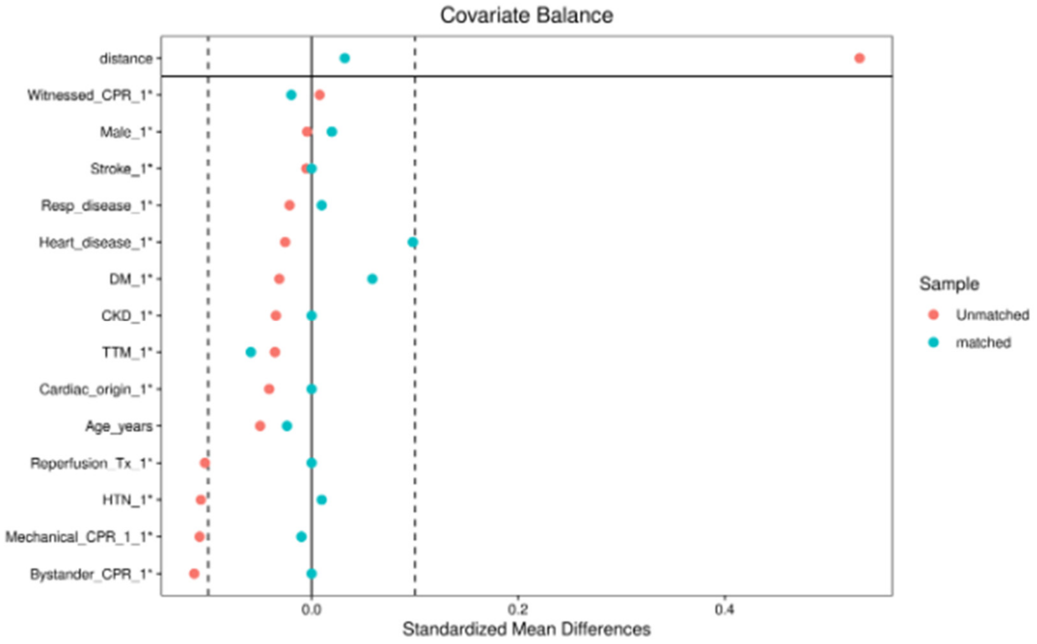

(B)

Figure S1. Changes in absolute standardized mean differences (A) and dot plot of absolute standardized mean differences (B) in ECPR patients before and after propensity score matching.

Abbreviations: CPR, cardiopulmonary resuscitation; CKD, chronic kidney disease; DM, diabetes mellitus; HTN, hypertension; TTM, targeted temperature management; ECPR, extracorporeal cardiopulmonary resuscitation.

Supplementary Table S1. Definition and detailed classification of pre-existing comorbidity

Disease	Definition and detailed classification
Hypertension	The disease was diagnosed by the doctor prior to cardiac arrest and is clearly stated in the medical record
Diabetes mellitus	The disease was diagnosed by the doctor prior to cardiac arrest and is clearly stated in the medical record.
Heart disease	The disease was diagnosed by the doctor prior to cardiac arrest and is clearly stated in the medical record. (Ischemic heart disease, myocardial infarction, angina, valvular heart disease, arrhythmia, congestive heart disease, myocardiopathy, congenital heart disease, previous heart intervention record including stent, primary coronary intervention, coronary artery bypass grafting, and other heart disease)
Chronic renal disease	The disease was diagnosed by the doctor prior to cardiac arrest and is clearly stated in the medical record. (chronic renal failure, dialysis, kidney transplantation, and other chronic kidney disease)
Chronic respiratory disease	The disease was diagnosed by the doctor prior to cardiac arrest and is clearly stated in the medical record. (Asthma, chronic obstructive pulmonary disease, pulmonary tuberculosis, other chronic respiratory disease.)
Stroke	The disease was diagnosed by the doctor prior to cardiac arrest and is clearly stated in the medical record. (cerebral infarction, cerebral haemorrhage, and unclassified stroke)
Dyslipidemia	The disease was diagnosed by the doctor prior to cardiac arrest and is clearly stated in the medical record. (hyperlipidaemia and unclassified dyslipidemia)

Supplementary Table S2. Glasgow-Pittsburgh Cerebral Performance Categories (CPC) scores and their corresponding descriptions

CPC Score	Description
CPC 1	Good cerebral performance with mild or no neurological deficit
CPC 2	Moderate cerebral disability with moderate neurological deficit, sufficient functional independence for activities of daily living
CPC 3	Severe cerebral disability with severe neurological deficit, dependence on others for daily activities
CPC 4	Coma or vegetative state, absence of meaningful neurological function, inability to interact with the environment
CPC 5	Brain death or death due to neurological injury
